# MOMENTARY ASSOCIATIONS BETWEEN PURPOSE IN LIFE AND COGNITIVE FUNCTION IN EVERYDAY LIFE

**DOI:** 10.1093/geroni/igad104.1028

**Published:** 2023-12-21

**Authors:** Angelina Sutin, Martina Luchetti, Alyssa Gamaldo, Jacqueline Mogle, Hephzibah Lovett, Justin Brown, Martin Sliwinski, Antonio Terracciano

**Affiliations:** Florida State University College of Medicine, Tallahassee, Florida, United States; Florida State University, College of Medicine, Tallahassee, Florida, United States; Clemson University, Clemson, South Carolina, United States; Clemson University, Clemson, South Carolina, United States; Florida State University College of Medicine, Tallahassee, Florida, United States; Florida State University College of Medicine, Tallahassee, Florida, United States; Pennsylvania State University, State College, Pennsylvania, United States; Florida State University, Tallahassee, Florida, United States

## Abstract

Purpose in life has been associated consistently with healthier cognitive aging outcomes, including better cognitive function and lower risk of incident dementia. Most within-person research on purpose and cognition has focused on cognition measured over years, which has been essential to identify the long-term protective benefits of purpose. The present research shrinks the timescale to momentary assessments over days to address whether purpose operates in the moment to support healthier cognitive function in everyday life. Participants from the Couples Healthy Aging Project (N=303) completed smartphone-based momentary assessments of purpose and short cognitive tasks three times a day for eight days. In moments when participants felt more purpose driven than their average, they had faster processing speed (b=-1.240, SE=0.194; p<.001) and less variability in processing speed (b=-0.437, SE=0.135; p<.001). These associations were independent of person, temporal, and contextual factors and practice effects. Momentary purpose was unrelated to visual memory performance. Similar patterns were found for other purpose-related items (momentary engagement, motivation) but not for hedonic emotions (momentary positive affect). These results suggest that feeling more purpose-driven in the moment supports faster processing speed in daily life. This work demonstrates that the positive association between purpose in life and better cognitive function operates in daily life and may be one mechanism through which purpose is associated with better long-term cognitive outcomes.

